# Trends in the incidence of the Epstein-Barr virus-associated malignancies extranodal NK/T-cell lymphoma and nasopharyngeal carcinoma in Taiwan

**DOI:** 10.1371/journal.pone.0315380

**Published:** 2024-12-31

**Authors:** Shyh-An Yeh, Liang-Chun Chiu, Hsin-Chieh Lin, Hung-Ju Li, Sheng-Fung Lin, Yu-Chieh Su

**Affiliations:** 1 Medical Physics and Informatics Laboratory of Electronic Engineering, National Kaohsiung University of Science and Technology, Kaohsiung, Taiwan; 2 Department of Electronic Engineering, National Kaohsiung University of Science and Technology, Kaohsiung, Taiwan; 3 Department of Medical Imaging and Radiological Sciences, I-Shou University, Kaohsiung, Taiwan; 4 Department of Radiation Oncology, E-DA Hospital, I-Shou University, Kaohsiung, Taiwan; 5 Department of Obstetrics and Gynecology, E-Da Dachang Hospital, I-Shou University, Kaohsiung, Taiwan; 6 Department of Chinese Medicine, E-Da Cancer Hospital, I-Shou University, Kaohsiung, Taiwan; 7 School of Chinese Medicine for Post-Baccalaureate, College of Medicine, I-Shou University, Kaohsiung, Taiwan; 8 Department of Internal Medicine, Division of Hematology-Oncology, E-Da Hospital, I-Shou University, Kaohsiung, Taiwan; 9 School of Medicine, College of Medicine, I-Shou University, Kaohsiung, Taiwan; 10 Graduate Institute of Medicine, College of Medicine, I-Shou University, Kaohsiung, Taiwan; Guangdong Medical University, CHINA

## Abstract

Nasopharyngeal carcinoma (NPC) and extranodal NK/T-cell lymphoma (ENKTL) are associated with the Epstein-Barr virus (EBV). The current study aimed to evaluate the trends in the incidence of ENKTL and NPC in Taiwan within the last 15–30 years. To assess the incidence of ENKTL from 2008 to 2021 and NPC from 1995 to 2021, an epidemiological study was performed using population-based registry data from the Taiwan Cancer Registry. The secular trends in the annual incidence rates were expressed as average annual percent change (AAPC). During the study period, 872 new ENKTL diagnoses and 39412 new NPC diagnoses were reported. The annual age-adjusted incidence rates of ENKTL and NPC decreased significantly, with an AAPC of −2.47 (*p* = 0.014) and −1.16 (*p* < 0.001), respectively. The incidence rates of NPC from 1995 to 2021 in the 20–44-, 45–64-, and ≥65-year-old age groups decreased from 5.23 to 4.42 per 100,000 person-years (AAPC = −0.45, *p* = 0.011), from 15.28 to 11.36 per 100,000 person-years (AAPC = −1.21, *p* < 0.001), and from 10.48 to 7.24 per 100,000 person-years (AAPC = −1.91, *p* < 0.001), respectively. The age-specific incidence rates of ENKTL in the 45–64-year-old age group significantly decreased from 0.46 to 0.32 per 100,000 person-years during 2008–2021 (AAPC = −3.37, *p* = 0.008). The incidence of ENKTL between 2008 and 2021 in the 20–44- and ≥65-year-old age groups also decreased from 0.16 to 0.11 per 100,000 person-years and from 0.87 to 0.79 per 100,000 person-years, respectively. Interestingly, the EBV seroprevalence in Taiwan remained stable from 1984 to 2007. Nonetheless, there was a downward trend in the EBV seroprevalence in early childhood, with a decrease of approximately 20% in children aged 4 years and nearly 50% in children aged <2 years. Based on these findings, the downward trend in the incidence of NPC/ENKTL could be affected by the decline in early EBV infection rates. Considering that the EBV seroprevalence remained stable but the incidence rate of early EBV infection decreased during the same period, delayed EBV infection or factors other than EBV might play an important role in the downward trend in the incidence of NPC/ENKTL in Taiwan. Nevertheless, further investigations should be performed to assess these results.

## Introduction

The Epstein-Barr virus (EBV), first discovered in 1964 by Epstein, Achong, and Barr in their study of Burkitt lymphoma, is the first human virus confirmed to be related to cancer pathogenesis [1]. EBV has been classified as a group 1 carcinogen by the International Agency for Research on Cancer. Additionally, it is associated with the development of several malignant tumors (including Burkitt lymphoma, Hodgkin lymphoma, diffuse large B-cell lymphoma, EBV-associated NK/T-cell lymphoma, stomach cancer, and nasopharyngeal carcinoma [NPC]) and related deaths [2]. More than 90% of the world’s population has been infected with EBV. However, in most cases, EBV remains latent in the human body and asymptomatic for life, and it can reactivate only in a few cases. Several clinical studies have shown that EBV can be activated by environmental factors in the environment, that is, enter the lytic stage. A large amount of viral DNA and proteins, which may be related to EBV pathogenicity, are produced during the lysis phase [3–5].

NPC is a special type of head and neck cancer, and its pathogenesis is extremely related to EBV. Although the global incidence rate of NPC is relatively low (1.3 per 100,000 persons)–with approximately 120,400 new cases diagnosed worldwide in 2020, accounting for only 0.6% of all cancers and ranking 23rd in terms of the incidence rate of all cancers–the disease exhibits a marked geographical distribution. Specifically, the incidence of NPC is significantly higher in Asia and North Africa, where age-standardized rates (ASRs) reach 1.8 and 1.7 per 100,000 persons, respectively [6]. In Asia, countries such as China and Taiwan have some of the highest incidence rates globally, with ASR ranging from 2.1 to 6.4 per 100,000 persons [7]. In Taiwan, the incidence rate of NPC in 2021 is approximately 4.30 per 100,000 persons. The incidence rate of NPC in male patients is approximately 3.5 times higher than that of female patients (6.75:1.94 per 100,000 persons) [8]. NPC is histologically classified into keratinizing, non-keratinizing (differentiated and undifferentiated) and basaloid squamous cell carcinomas. Non-keratinizing undifferentiated carcinoma is the most common type in areas with high incidence rates of NPC. The prevalence rate of EBV in non-keratinizing carcinoma NPC was 100%. In 2021, 96% of NPC cases in Taiwan were non-keratinizing squamous cell carcinoma. Based on this finding, EBV is extremely related to NPC pathogenesis.

Nasal-type extranodal NK/T-cell lymphoma (ENKTL) is another type of malignancy whose pathogenesis is closely related to EBV. Almost all patients with ENKTL are EBV-positive, and ENKTL is a rare malignancy with significant racial and geographic variations worldwide [9]. The frequency of ENKTL was higher in Asian countries than in Western countries and in Continental Asian countries than in Japan [10]. ENKTL accounts for 4.2%, 1.2%, 1.6%, 0.1%, and 0.1% of all lymphomas in South Korea, Japan, Singapore, the USA, and the UK, respectively [11–15]. In Taiwan, ENKTL is the second most common T-cell lymphoma, accounting for 2.7% of all lymphomas [16].

The pathogenesis of NPC and ENKTL is extremely related to EBV. Hence, the incidence rates of these two conditions in Taiwan are higher than those in other countries. Previous studies have shown that the incidence rate of NPC in Taiwan has a downward trend from 1981 to 2000, and this finding can be attributed to lifestyle changes. The incidence rate of ENKTL in Taiwan has an upward trend from 2002 to 2012. However, previous studies showed a downward trend from 2008 to 2020 [17–19]. Therefore, the current study aimed to assess the trends in the incidence rate of NPC and ENKTL in recent years using data from a population-based cancer registry in Taiwan. Furthermore, the association between NPC and ENKTL and EBV seroprevalence was explored.

## Materials and methods

### Data sources

This study analyzed patients with NPC who were newly diagnosed from 1995 to 2021 and patients with ENKTL who were newly diagnosed from 2008 to 2021. Population-based data were obtained from the Cancer Registry Annual Report of the Health Promotion Administration, Ministry of Health and Welfare, Taiwan [20]. These annual reports were based on the data from the Taiwan Cancer Registry (TCR) database, a national population-based cancer registry system with high-quality registry indicators. The time frame for NPC was selected based on the availability of consistent annual data in the TCR since 1995, while ENKTL data were only available from 2008 onward in the annual reports. Data quality indicators are mainly used to evaluate the completeness and validity of the registration system. The International Agency for Research on Cancer uses the percentage of death certificate-only cases (DCO%) as an indicator of completeness, and the percentage of morphologically verified cases (MV%) as an indicator of validity. The completeness rate of the cancer cases in the TCR ranged from 91.3% in 2001 to 98.1% in 2020. The DCO% decreased from 8.84% in 1998 to 0.77% in 2021. The MV% ranges from 87.5% in 2002 to 94.5% in 2021.

### Data analysis

The International Classification of Disease for Oncology was used to identify patients with NPC (ICD-O-FT code 147 in 1995–2001/ICD-O-3 code C11 in 2002–2021) and those with ENKTL (ICD-O-3 code 9719). The data used in this study are publicly available and do not include any patient identification information. Hence, the current study did not require approval from the institutional review board.

The incidence of malignancy was described using the crude incidence rate (CR), age-specific incidence rate, and ASR, all expressed per 100,000 person-years. To prevent low counts at extreme ages, age-specific incidence rates were calculated based on three age groups (20–44, 45–64, and ≥65 years). Annual percentage changes (APCs) and average annual percent changes (AAPCs) were used to characterize the secular patterns in the annual incidence rates. Using the year as a regressor variable and fitting a least squares regression line to the natural logarithm of the incidence rates, the APC was confirmed. The line’s slope and standard error were used to calculate the point estimate and 95% confidence interval of the APC.

Analyses were conducted using the Joinpoint Regression Program (Joinpoint version 4.9.1.0; NCI Statistical Methodology and Applications Branch, Bethesda, MD, USA) [21]. An independent two-sided t-test was conducted to determine whether the APC was statistically significant from 0. A *p*-value of <0.05 indicated statistically significant differences.

## Results

### Trends in the incidence of NPC and ENKTL

**Fig 1:**
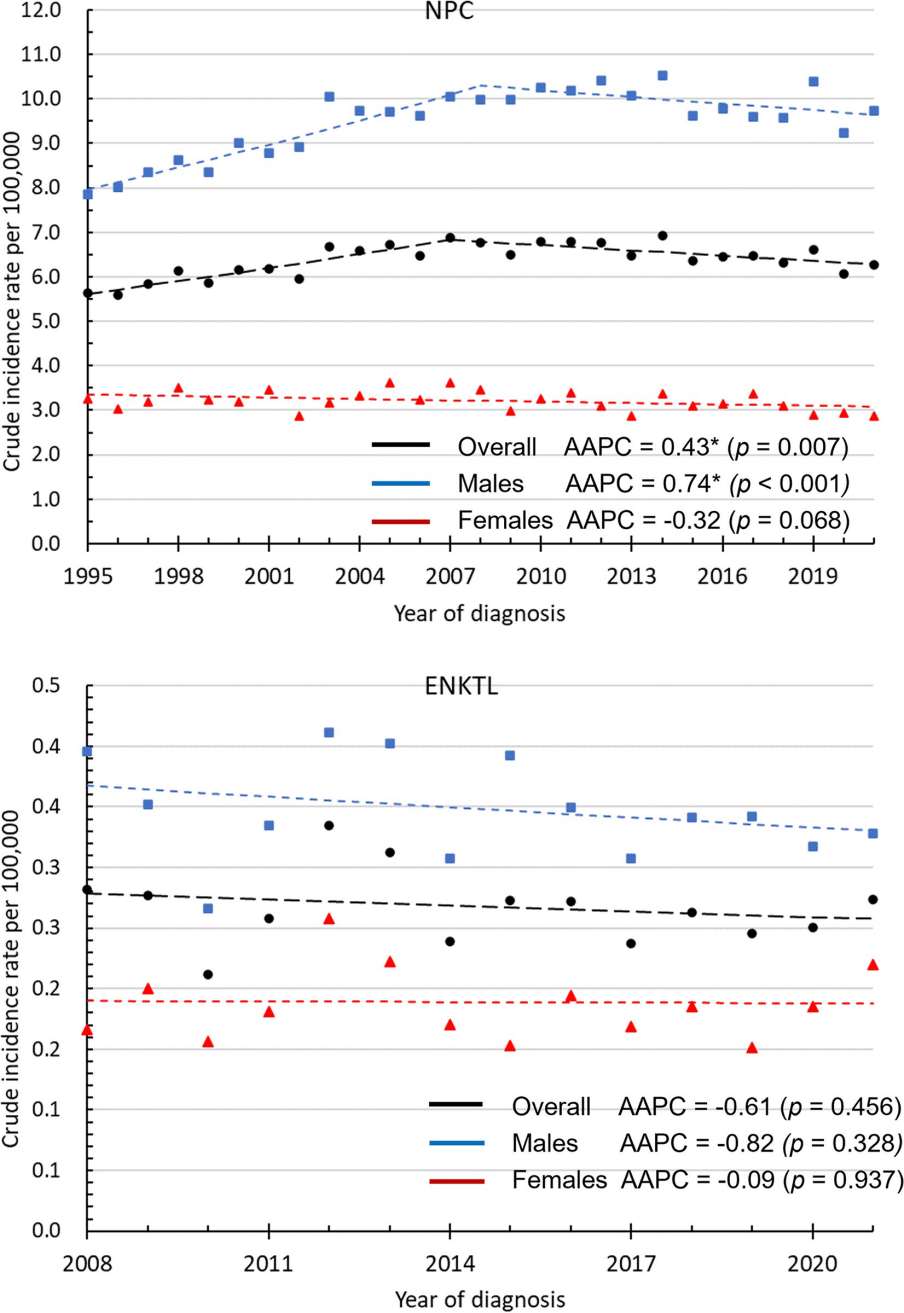
The crude incidence rates of NPC and ENKTL. *The AAPC is significantly different from 0 at the alpha level of 0.05.

**Fig 2:**
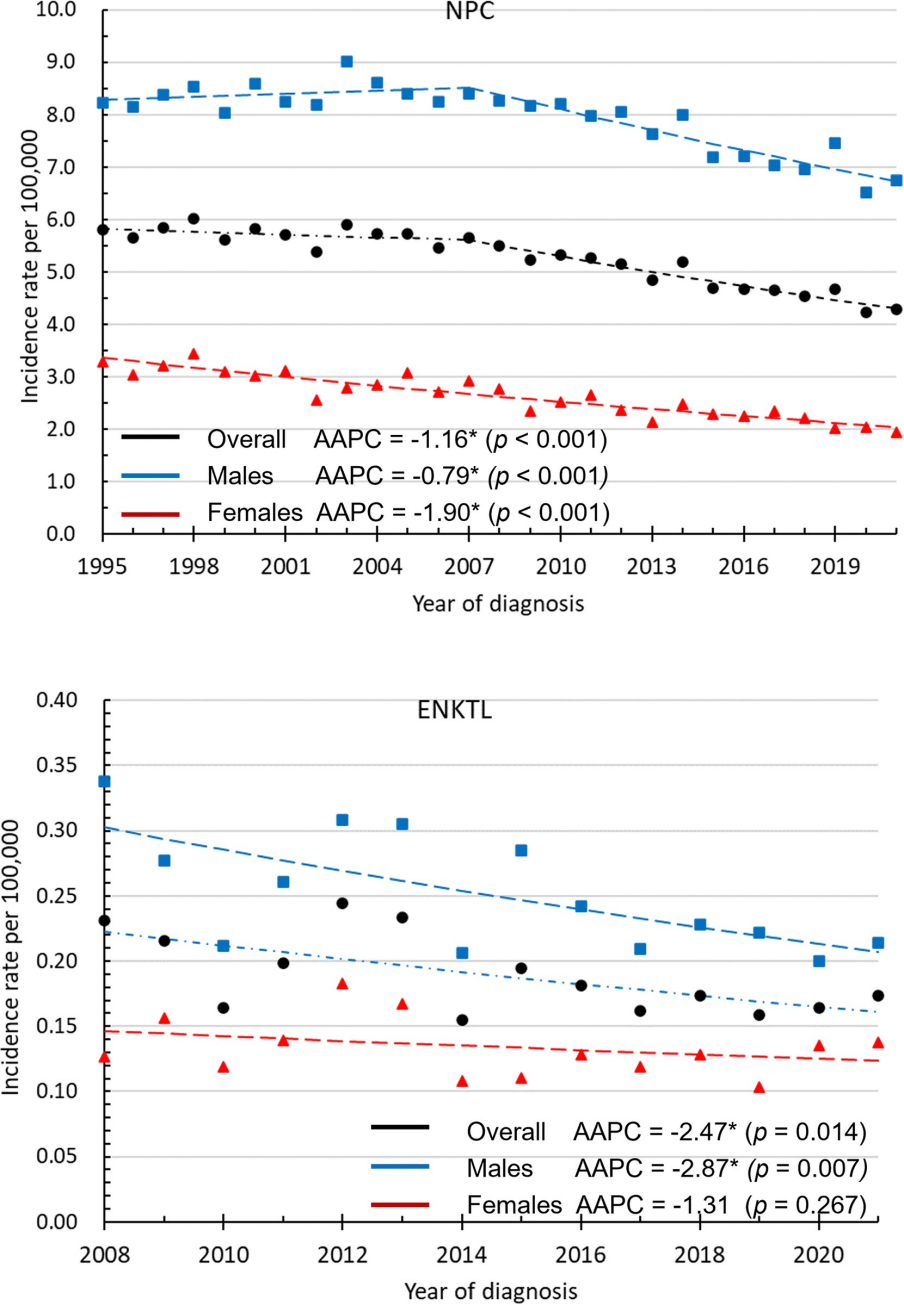
The age-standardized incidence rates of NPC and ENKTL. *The AAPC is significantly different from 0 at the alpha level of 0.05.

In total, 39,412 patients, including 29,608 (75.1%) men and 9,804 (24.9%) women, were diagnosed with NPC between 1995 and 2021. Furthermore, 872 patients, including 566 (64.9%) men and 306 (35.1%) women, were diagnosed with ENKTL between 2008 and 2021. Figs 1 and 2 show the CRs and age-standardized incidence rates of NPC and ENKTL.

As depicted in Fig 1, the CR of NPC increased from 5.65 per 100,000 person-years in 1995 to 6.27 per 100,000 person-years in 2021, with an AAPC of 0.43 (95% CI: 0.12–0.74, *p* = 0.007). The CR of NPC in male patients increased from 7.85 per 100,000 person-years in 1995 to 9.73 per 100,000 person-years in 2021, with an AAPC of 0.74 (95% CI: 0.39–1.08, *p* < 0.001). Meanwhile, the CR of NPC in female patients decreased from 3.35 per 100,000 person-years in 1995 to 3.08 per 100,000 person-years in 2021, with an AAPC of −0.32 (95% CI: −0.67 to 0.03, *p* = 0.068). The CR of ENKTL decreased from 0.28 per 100,000 person-years in 2008 to 0.27 per 100,000 person-years in 2021, with an AAPC of −0.61 (95% CI: −2.3 to 1.12, *p* = 0.456). In male patients, the CR of ENKTL decreased from 0.40 per 100,000 person-years in 2008 to 0.33 per 100,000 person-years in 2021. However, there was no evident change in the CR of ENKTL in female patients, which ranged from 0.17 per 100,000 person-years in 2008 to 0.22 per 100,000 person-years in 2021. S1 and S2 Tables present the detailed annual incidence rates of NPC and ENKTL.

Fig 2 shows the secular trends in the ASRs of NPC and ENKTL. The ASR of NPC had significantly downward trends in the overall cohort, male patients, and female patients in Taiwan, with APCs of −1.16 (95% CI: −1.47 to −0.86), −0.79 (95% CI: −1.14 to −0.45), and −1.90 (95% CI: −2.21 to −1.58), respectively. The ASR of NPC from 1995 to 2021 declined by 26.2%, from 5.81 to 4.29 per 100,000 person-years in the overall cohort. The ASR of NPC in male patients decreased by 18.1%, from 8.23 to 6.74 per 100,000 person-years. The ASR of NPC in female patients decreased by 41.1%, from 3.29 to 1.94 per 100,000 person-years. The ASR of ENKTL in the overall cohort and male patients had a significantly downward trend. However, the ASR of ENKTL in female patients remained stable. The ASR of ENKTL decreased by 24.8%, from 0.23 to 0.17 per 100,000 person-years (AAPC = −2.47, 95% CI: −4.29 to −0.61, *p* = 0.14), in the overall cohort. In male patients, the ASR of ENKTL decreased by 36.6%, from 0.34 to 0.21 per 100,000 person-years (AAPC = −2.87, 95% CI: −4.78 to −0.93, *p* = 0.007). In female patients, the ASR of ENKTL ranged from 0.10 to 0.18 per 100,000 person-years (AAPC = −1.31, 95% CI: −3.70 to 1.15, *p* = 0.267).

**Fig 3:**
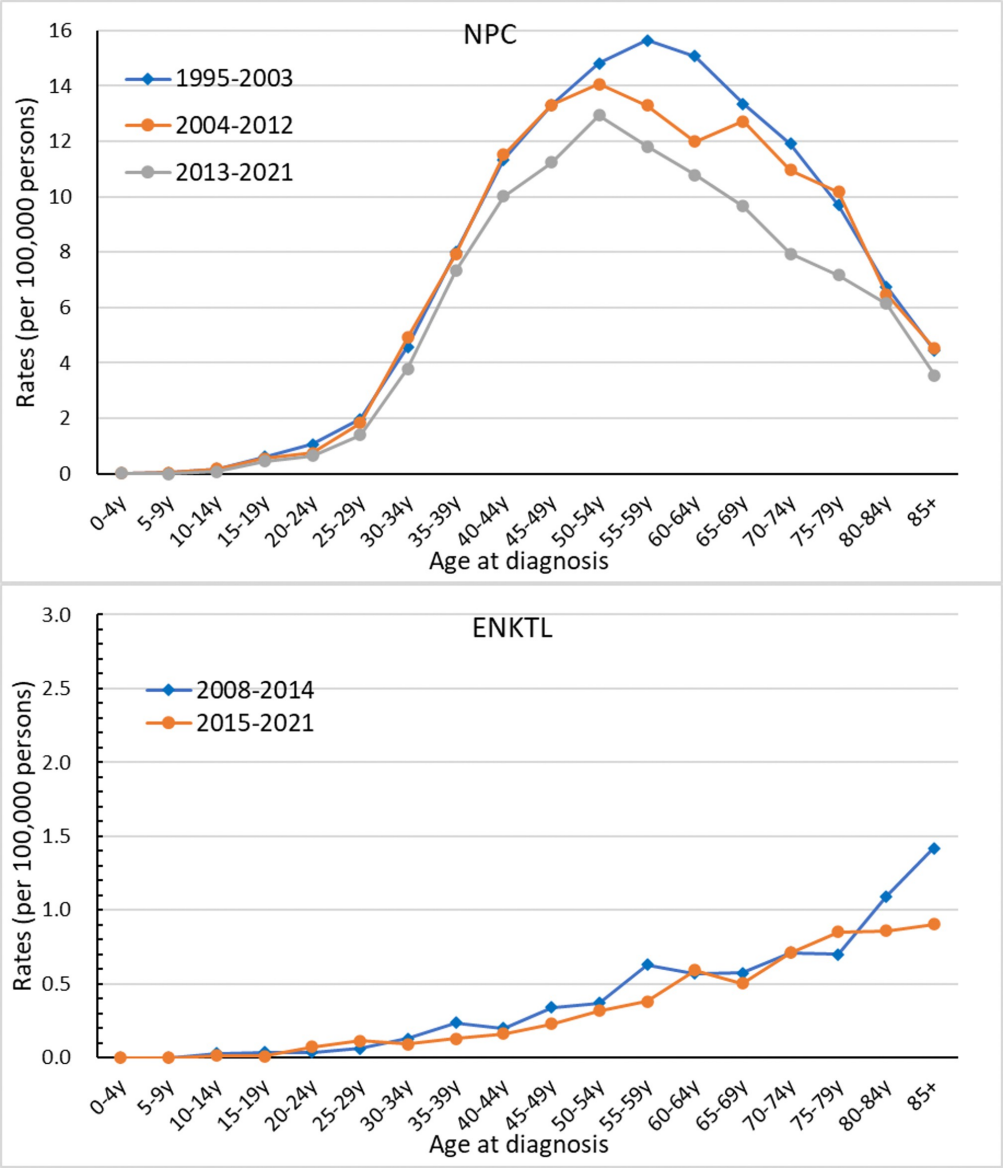
The age-specific incidence rates of NPC and ENKTL across different time-periods.

**Fig 4:**
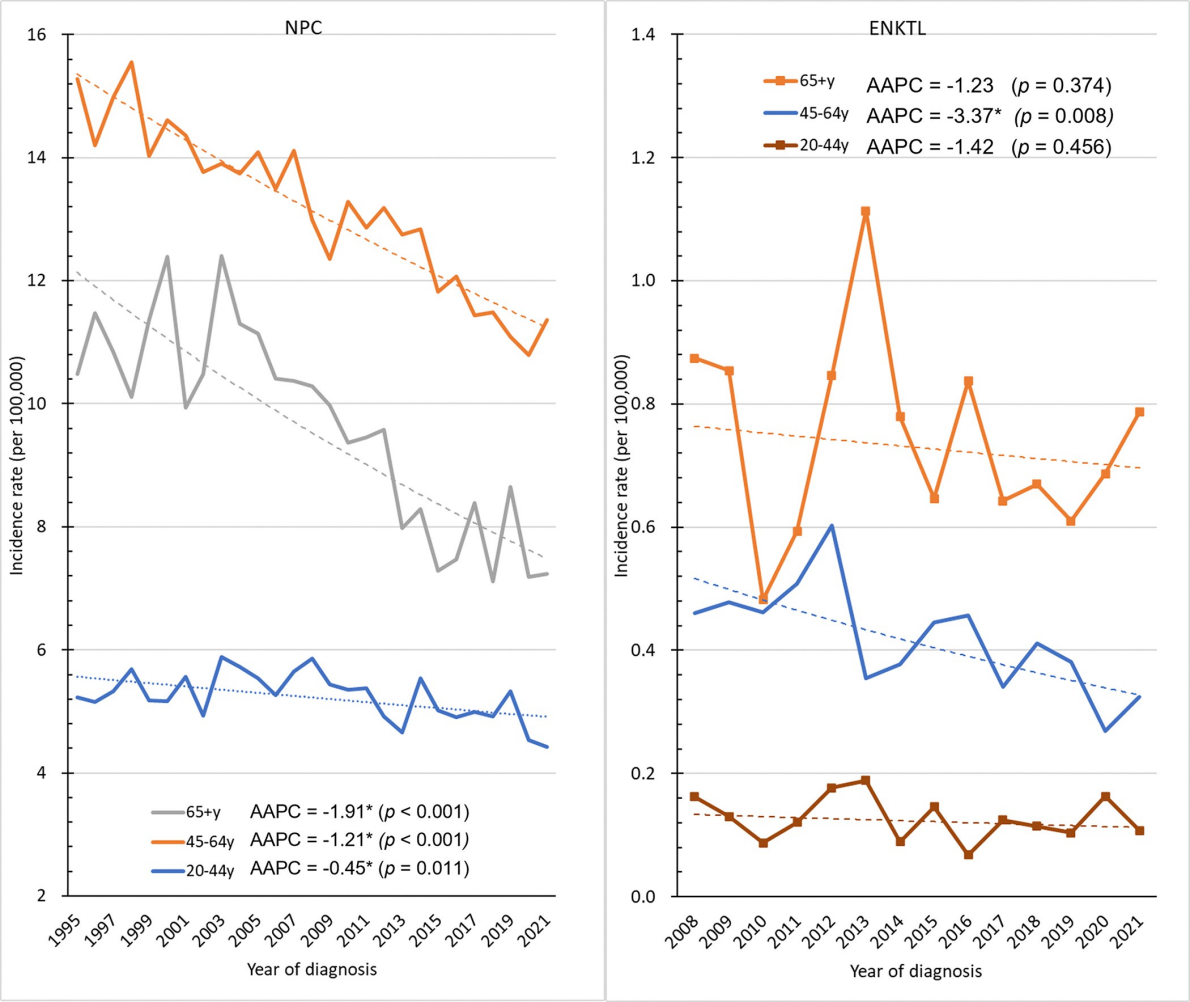
Trends in age-specific incidence rates of NPC and ENKTL. *The AAPC is significantly different from 0 at the alpha level of 0.05.

Fig 3 shows the age-specific incidence rates of NPC and ENKTL among different time periods. From 1995 to 2003, the age-specific incidence rates of NPC peaked at 55–59 years of age. Since 2004, the incidence rates of NPC had peaked at 50–54 years of age. In all age groups, the age-specific rates of NPC during 2013–2021 was lower than that during 1995–2003. The age-specific incidence rates of ENKTL increased with age. Similar to NPC, the age-specific incidence rate of ENKTL during 2015–2021 was lower than that during 2008–2014 in all age groups. Fig 4 shows the annual trends in the age-specific incidence rates in different age groups—20–44 years, 45–64 years, and ≥65 years. The incidence rate of NPC in the 20–44-, 45–64-, and ≥65-year-old age groups decreased from 5.23 per 100,000 person-years in 1995 to 4.42 per 100,000 person-years in 2021 (AAPC = −0.45, *p* = 0.011), from 15.28 per 100,000 person-years in 1995 to 11.36 per 100,000 person-years in 2021 (AAPC = −1.21, *p* < 0.001), and from 10.48 per 100,000 person-years in 1995 to 7.24 per 100,000 person-years in 2021 (AAPC = −1.91, *p* < 0.001), respectively. In the 45–64-year-old age group, the age-specific incidence rates of ENKTL significantly decreased from 0.46 per 100,000 person-years in 2008 to 0.32 per 100,000 person-years in 2021 (AAPC = −3.37, *p* = 0.008). However, the age-specific incidence rates of ENKTL in the 20–44- and ≥65-year-old age groups decreased from 0.16 per 100,000 person-years in 2008 to 0.11 per 100,000 person-years in 2021 and from 0.87 per 100,000 person-years in 2008 to 0.79 per 100,000 person-years in 2021, respectively. Hence, the results did not significantly differ. Nevertheless, the average number of patients in the 20–44- and ≥65-year-old age groups was relatively small (11.4 and 22.2, respectively).

### EBV seroprevalence in Taiwan

Previous studies on EBV seroprevalence in Taiwan are extremely rare. There are only two recorded studies: one in 1989, which focused on 1,350 children aged <15 years in Taipei [21], and the other in 2007, which used samples from a large public health survey that comprised 1,411 human serum samples and covered all age categories and areas in Taiwan [22]. According to the findings of these two studies, the EBV seroprevalence rate in infants aged <1 year was 22%–24%. In the 1-year-old age group, the EBV seroprevalence rates were 65.8% in 1984 and 30.0% in 2007. In the 4-year-old age group, the EBV seroprevalence rates were 86% in 1984 and 69.1% in 2007. In the 10–19-year-old age group, the EBV seroprevalence rates were 93% in 2007 and 99% in 1984. The EBV seroprevalence in Taiwan remained stable from 1984 to 2007, with a rate of >90% among individuals aged >10 years. Nonetheless, the EBV seroprevalence in early childhood had a declining trend, with a reduction in the prevalence by approximately 20% in children aged 4 years and by almost 50% in children aged <2 years (S3 Table).

## Discussion

This study used population-based data to assess the trend in the incidence of NPC from 1995 to 2021 and ENKTL from 2008 to 2021 in Taiwan. Results showed that the incidence rates of NPC and ENKTL, whose pathogenesis is closely related to EBV, exhibited a significantly downward trend. Additionally, the trend in the age-specific incidence rate of NPC significantly decreased in three different age groups (20–44, 45–64, and ≥ 65 years). The incidence of ENKTL exhibited a significantly downward trend in the 45–64-year-old age group alone. Meanwhile, the incidence of ENKTL in the 20–44- and ≥65-year-old age groups had a non-significant downward trend. This may be attributed to the small number of cases, with an annual average of 11 and 22 in the 20–44- and ≥65-year-old age groups, respectively. To gain insight about the trend in the EBV seroprevalence in Taiwan, the studies of Tsai et al. (1989) and Chen et al. (2007) were examined [22,23]. These studies showed that the EBV seroprevalence in Taiwan remained stable from 1984 to 2007, with a rate of >90% in individuals aged >10 years. This result indicates that the downward trend in the incidence of NPC and ENKTL is not attributed to the decrease in EBV infection rates.

There is no downward trend in the EBV infection rate. However, in most cases, EBV will remain latent in the human body without symptoms. Moreover, it will only produce a large amount of viral DNA and proteins after reactivation, which trigger the development of related malignant tumors. EBV reactivation is related to environmental factors, and smoking is a possible influencing factor [4,24].

According to the results of the Adult Smoking Behavior Survey (ASBS) by the Taiwan Health Promotion Administration, Ministry of Health and Welfare, over the years, since the implementation of the Tobacco Harm Prevention Act in 1996, which was driven by various policies, the smoking rate among adults has decreased from 29.2% in 1996 to 13.1% in 2020. Furthermore, the smoking rate among men reduced from 55.1% in 1996 to 23.1% in 2020 [25]. Therefore, it is hypothesized that with the reduction in the proportion of the smoking population, the incidence of EBV activation will also decrease. Nevertheless, EBV reactivation can only be identified via epidemiological surveys and tests using blood or other samples. Previous studies on this notion are extremely limited. K. Takeuchi et al. evaluated the trend in EBV seroprevalence among children aged 5–7 years in Tokyo, Japan. From 1975 to 1979, >85% of children aged 5–7 years were serologically positive for EBV. However, during 1995–1999, the positivity rate decreased to 59% [26]. Based on a retrospective study that assessed 10,560 blood samples from Bahrain during 2001–2015, the EBV reactivation rate decreased from 21.1% in 2001 to 3.3% in 2015. In the >25-year-old age group, the reactivation rate decreased significantly from 53.2% to 42.9% during the study period [27].

Other environmental factors that increase the risk of EBV activation include the use of household insecticides and pesticides. A previous report revealed that during environmental or occupational exposure, pesticides may temporarily or permanently alter the immune system, thereby increasing the risk of EBV reactivation [28]. According to previous research, pesticides such as atrazine and pyrethroids inhibit the survival and growth of white blood cells by inducing apoptosis or cell cycle arrest and by interfering with the specific immune functions of each type of immune cell [29]. According to an exploratory prospective analysis of three agricultural cohorts who participated in the AGRICOH consortium, Hodgkin lymphoma, a type of cancer related to EBV, may be associated with pesticides such as pyrethroids [30]. A population-based case-control study in Sweden revealed that insecticide exposure was associated with a slight increase in the odds ratio of non-Hodgkin lymphoma [31]. Within the last 20 years, Taiwan has made significant progress in reducing pesticide use. The government began actively establishing Taiwan’s sustainable development indicators (TSDI) system in 2003 to promote organic agriculture and integrated pest control strategies to reduce pesticide residues and shift to more sustainable practices. The total area of organically certified cropping in Taiwan has increased from 900 hectares in 2001 to approximately 19,000 hectares in June 2024 [32,33]. Hence, the use of pesticides in agricultural settings has significantly decreased. There are no relevant studies on the trends in household insecticide use. Nevertheless, the overall trend reflects people’s increasing knowledge on environment and health, which may lead to households becoming more concerned about the use of household pesticides. The reduced reliance on insecticides further decreases the risk of EBV activation caused by insecticide factors.

Our research findings also clearly reflected a significant sex difference in the incidence of NPC and ENKTL, with the incidence of NPC in men about three times that in women, and the incidence of ENKTL in men about twice that in women. However, since EBV reactivation is linked to environmental factors such as smoking—where male smoking rates are 7–8 times higher than females in Taiwan in 2020—environmental exposure may also play a role [25]. Hu et al. (2019) observed an interaction between smoking and sex on EBV reactivation, with female smokers having a higher NPC risk than male smokers, though their overall NPC incidence remained lower due to low smoking rates [34]. Mokarizadeh et al. (2015) explained that sex could influence sensitivity to environmental exposures through metabolic rate and baseline immune responses [28]. Therefore, more investigation is required into how sex and environmental factors combine to cause EBV reactivation.

Early EBV infection is also a risk factor of NPC and NK/T-cell lymphomas. It has been proposed that hereditary transmission and inadequate personal hygiene can lead to early-life EBV infection [35]. In contrast to the findings of Tsai et al. in 1989 and Chen et al. in 2007, the early EBV infection rate in Taiwan may have decreased [22,23]. This can be one of the causes of the decline in the incidence of NPC and ENKTL. Hence, further investigations must be performed to assess this finding.

This study had some limitations. First, the trend in the incidence of ENKTL was assessed only from 2008 to 2021 because the TCR Annual Report did not include ENKTL-specific data prior to 2008. Additionally, the analysis relied on the NPC data from 1995 to 2021, as TCR data has been available for this cancer type since 1995. Second, while EBV seroprevalence in Taiwan was estimated using two previous studies, the years of serum collection for these studies were 1984 and 2007, which were before the research periods for NPC and ENKTL incidence. This time frame discrepancy may limit the direct comparability of EBV seroprevalence trends with the observed trends in NPC and ENKTL incidence. Future studies would benefit from updated seroprevalence data that align more closely with the incidence data.

## Conclusions

The trends in the incidence of NPC and ENKTL in Taiwan have been decreasing within the last 20 years. In the same period, the EBV seroprevalence remained stable, and the incidence of early EBV infection decreased. These findings indicate that delayed primary EBV infection is associated with a decline in the incidence of NPC and ENKTL or other factors such as a decrease in smoking rates and pesticide use may contribute to the progressive reduction in the incidence of NPC and ENKTL. Nevertheless, further research must be conducted to confirm the findings of this study.

## Supporting information

S1 Table Number of incidence cases, crude incidence rates (CRs) and age-standardized rates (ASRs) of patients with NPC between 1995 and 2021. (DOCX)

S2 Table Number of incidence cases, crude incidence rates (CRs) and age-standardized rates (ASRs) of patients with ENKTL between 2008 and 2021. (DOCX)

S3 Table Seroprevalence of EBV in Taiwan. (DOCX)

## References

[1] Epstein MA, Achong BG, Barr YM. Virus particles in cultured lymphoblasts from Burkitt’s lymphoma. The Lancet. 1964;283(7335):702–3. doi: 10.1016/s0140-6736(64)91524-7 14107961

[2] IARC Working Group on the Evaluation of Carcinogenic Risks to Humans. Epstein-Barr Virus and Kaposi’s Sarcoma Herpesvirus/Human Herpesvirus 8. Lyon (FR): International Agency for Research on Cancer; 1997. (IARC Monographs on the Evaluation of Carcinogenic Risks to Humans, No. 70.) Epstein-Barr virus. Available from: https://www.ncbi.nlm.nih.gov/books/NBK385500/. PMC5046075 9705682

[3] Mydin RBS, Okekpa SI. Molecular Pathways for Nasopharyngeal Carcinoma focused on Acetaldehyde, Nitrosamines and Nicotine Exposures. Malaysian Journal of Medicine & Health Sciences. 2019;15(SP2):64–70.

[4] Chen Y, Chang ET, Liu Q, Cai Y, Zhang Z, Chen G, et al. Environmental Factors for Epstein-Barr Virus Reactivation in a High-Risk Area of Nasopharyngeal Carcinoma: A Population-Based Study. Open Forum Infect Dis. 2022;9(5):ofac128. doi: 10.1093/ofid/ofac128 35450082 PMC9017372

[5] Dickerson F, Katsafanas E, Origoni A, Newman T, Rowe K, Ziemann RS, et al. Cigarette smoking is associated with Herpesviruses in persons with and without serious mental illness. PLoS One. 2023;18(1):e0280443. doi: 10.1371/journal.pone.0280443 36652488 PMC9847975

[6] Bray F, Laversanne M, Sung H, Ferlay J, Siegel RL, Soerjomataram I, et al. Global cancer statistics 2022: GLOBOCAN estimates of incidence and mortality worldwide for 36 cancers in 185 countries. CA Cancer J Clin. 2024;74(3):229–63. doi: 10.3322/caac.21834 38572751

[7] Ferlay J, Ervik M, Lam F, Laversanne M, Colombet M, Mery L, et al. Global Cancer Observatory: Cancer Today: Lyon, France: International Agency for Research on Cancer; 2024. Available from: https://gco.iarc.who.int/today.

[8] Health Promotion Administration, Ministry of Health and Welfare, Taiwan. Cancer Registry Annual Report, 2021, Taiwan. Available from: https://www.hpa.gov.tw/File/Attach/17639/File_23506.pdf, released Nov 2023.

[9] Aozasa K, Takakuwa T, Hongyo T, Yang WI. Nasal NK/T-cell lymphoma: epidemiology and pathogenesis. Int J Hematol. 2008;87(2):110–7. doi: 10.1007/s12185-008-0021-7 18256789 PMC2276242

[10] Au WY, Weisenburger DD, Intragumtornchai T, Nakamura S, Kim WS, Sng I, et al. Clinical differences between nasal and extranasal natural killer/T-cell lymphoma: a study of 136 cases from the International Peripheral T-Cell Lymphoma Project. Blood. 2009;113(17):3931–7. doi: 10.1182/blood-2008-10-185256 19029440

[11] Sim J, Takayama T, Cho J, Kim SJ, Kim WS, Ree HJ, et al. Changing trends in lymphoid neoplasm distribution in South Korea: analysis of 8615 cases from a single institute, 1997–2016: An observational study. Medicine (Baltimore). 2019;98(45):e17641. doi: 10.1097/MD.0000000000017641 31702615 PMC6855639

[12] Chihara D, Ito H, Matsuda T, Shibata A, Katsumi A, Nakamura S, et al. Differences in incidence and trends of haematological malignancies in Japan and the United States. Br J Haematol. 2014;164(4):536–45. doi: 10.1111/bjh.12659 24245986 PMC3907701

[13] Lim RBT, Loy EY, Lim GH, Zheng H, Chow KY, Lim ST. Gender and ethnic differences in incidence and survival of lymphoid neoplasm subtypes in an Asian population: Secular trends of a population-based cancer registry from 1998 to 2012. 2015;137(11):2674–87. doi: 10.1002/ijc.29635 26061168

[14] Teras LR, DeSantis CE, Cerhan JR, Morton LM, Jemal A, Flowers CR. 2016 US lymphoid malignancy statistics by World Health Organization subtypes. CA Cancer J Clin. 2016;66(6):443–59. doi: 10.3322/caac.21357 27618563

[15] Smith A, Roman E, Howell D, Jones R, Patmore R, Jack A, et al. The Haematological Malignancy Research Network (HMRN): a new information strategy for population based epidemiology and health service research. Br J Haematol. 2010;148(5):739–53. doi: 10.1111/j.1365-2141.2009.08010.x 19958356 PMC3066245

[16] Chuang SS, Chen SW, Chang ST, Kuo YT. Lymphoma in Taiwan: Review of 1347 neoplasms from a single institution according to the 2016 Revision of the World Health Organization Classification. J Formos Med Assoc. 2017;116(8):620–5. doi: 10.1016/j.jfma.2016.11.006 28003113

[17] Hsu C, Shen YC, Cheng CC, Hong RL, Chang CJ, Cheng AL. Difference in the incidence trend of nasopharyngeal and oropharyngeal carcinomas in Taiwan: implication from age-period-cohort analysis. Cancer Epidemiol Biomarkers Prev. 2006;15(5):856–61. doi: 10.1158/1055-9965.EPI-05-0821 16702360

[18] Ko BS, Chen LJ, Huang HH, Wen YC, Liao CY, Chen HM, et al. Subtype-specific epidemiology of lymphoid malignancies in Taiwan compared to Japan and the United States, 2002–2012. Cancer Med. 2018;7(11):5820–31. doi: 10.1002/cam4.1762 30460792 PMC6246924

[19] Huang C-I, Ker C-Y, Li H-J, Hsiao Y-T, Lin S-F, Su Y-C. Incidence trends for common subtypes of T-cell lymphoma in Taiwan and the United States from 2008–2020. International Journal of Hematology. 2024;119:728–35. doi: 10.1007/s12185-024-03746-8 38494548

[20] Cancer registry annual report, Taiwan. Published by Health Promotion Administration, Ministry of Health and Welfare, Taiwan. (https://www.hpa.gov.tw/269/s), released Nov 2023.

[21] Joinpoint Regression Program, Version 4.9.1.0. April, 2022; Statistical Research and Applications Branch, National Cancer Institute.

[22] Tsai W, Chang M, Chen J-Y, Lee C, Liu Y-G. Seroepidemiological study of Epstein-Barr virus infection in children in Taipei. Zhonghua Min Guo Xiao Er Ke Yi Xue Hui Za Zhi. 1989;30(2):81–6. 2561579

[23] Chen CY, Huang KY, Shen JH, Tsao KC, Huang YC. A large-scale seroprevalence of Epstein-Barr virus in Taiwan. PLoS One. 2015;10(1):e0115836. doi: 10.1371/journal.pone.0115836 25615611 PMC4304788

[24] Xu FH, Xiong D, Xu YF, Cao SM, Xue WQ, Qin HD, et al. An epidemiological and molecular study of the relationship between smoking, risk of nasopharyngeal carcinoma, and Epstein-Barr virus activation. Journal of the National Cancer Institute. 2012;104(18):1396–410. doi: 10.1093/jnci/djs320 22972969

[25] Adult Smoking Behavior Survey (ASBS), published by Health Promotion Administration, Ministry of Health and Welfare, Taiwan. Available at https://www.hpa.gov.tw/1718/9913/n (last accessed 10.07.24) [updated 2024/06/25.

[26] Takeuchi K, Tanaka-Taya K, Kazuyama Y, Ito YM, Hashimoto S, Fukayama M, et al. Prevalence of Epstein–Barr virus in Japan: Trends and future prediction. Pathology International. 2006;56(3):112–6. doi: 10.1111/j.1440-1827.2006.01936.x 16497243

[27] Farid E, Al-Biltagi M. Trend and seroprevalence of Epstein-Barr virus in Bahrain: 2001–2015. East Mediterr Health J. 2018;23(12):821–9. doi: 10.26719/2017.23.12.821 29528092

[28] Mokarizadeh A, Faryabi MR, Rezvanfar MA, Abdollahi M. A comprehensive review of pesticides and the immune dysregulation: mechanisms, evidence and consequences. Toxicology Mechanisms and Methods. 2015;25(4):258–78. doi: 10.3109/15376516.2015.1020182 25757504

[29] Lee G-H, Choi K-C. Adverse effects of pesticides on the functions of immune system. Comparative Biochemistry and Physiology Part C: Toxicology & Pharmacology. 2020;235:108789. doi: 10.1016/j.cbpc.2020.108789 32376494

[30] Kim J, Leon ME, Schinasi LH, Baldi I, Lebailly P, Freeman LEB, et al. Exposure to pesticides and risk of Hodgkin lymphoma in an international consortium of agricultural cohorts (AGRICOH). Cancer Causes & Control. 2023;34(11):995–1003. doi: 10.1007/s10552-023-01748-1 37418114 PMC10533587

[31] Eriksson M, Hardell L, Carlberg M, Åkerman M. Pesticide exposure as risk factor for non-Hodgkin lymphoma including histopathological subgroup analysis. International Journal of Cancer. 2008;123(7):1657–63. doi: 10.1002/ijc.23589 18623080

[32] Tsai W-T. Analysis of coupling the pesticide use reduction with environmental policy for agricultural sustainability in Taiwan. Environment and Pollution. 2013;2(2):59. doi: 10.5539/ep.v2n2p59

[33] Yearly Report of Taiwan’s Agriculture: Ministry of Agriculture, Taiwan; [updated 2024/06. Available from: https://www.afa.gov.tw/cht/index.php?code=list&ids=563.

[34] Hu T, Lin CY, Xie SH, Chen GH, Lu YQ, Ling W, et al. Smoking can increase nasopharyngeal carcinoma risk by repeatedly reactivating Epstein‐Barr Virus: An analysis of a prospective study in southern China. Cancer medicine. 2019;8(5):2561–71. doi: 10.1002/cam4.2083 30843658 PMC6536979

[35] Poh SS, Chua MLK, Wee JT. Carcinogenesis of nasopharyngeal carcinoma: an alternate hypothetical mechanism. Chinese Journal of Cancer. 2016;35:1–9. doi: 10.1186/s40880-015-0068-9 26738743 PMC4704291

