# Supplementary material for: Trends in the incidence of the Epstein-Barr virus-associated malignancies extranodal NK/T-cell lymphoma and nasopharyngeal carcinoma in Taiwan
Source: PLoS One. 2024 Dec 31;19(12):e0315380. doi: 10.1371/journal.pone.0315380 (PMC11687711; doi:10.1371/journal.pone.0315380)
Supplement: S1 Table — (DOCX) [file pone.0315380.s001.docx]

S1 Table. Number of incidence cases, crude incidence rates (CRs) and age-standardized rates (ASRs) of patients with NPC between 1995 and 2021.

|  |  | Overall |  |  | Males |  |  | Females |  |
| --- | --- | --- | --- | --- | --- | --- | --- | --- | --- |
| Year | Number of  incidences | CR | ASR | Number of  incidences | CR | ASR | Number of  incidences | CR | ASR |
| 1995 | 1202 | 5.63 | 5.81 | 863 | 7.85 | 8.23 | 339 | 3.27 | 3.29 |
| 1996 | 1204 | 5.59 | 5.65 | 886 | 8.01 | 8.16 | 318 | 3.04 | 3.04 |
| 1997 | 1270 | 5.84 | 5.85 | 932 | 8.35 | 8.38 | 338 | 3.19 | 3.20 |
| 1998 | 1347 | 6.14 | 6.01 | 971 | 8.64 | 8.54 | 376 | 3.52 | 3.44 |
| 1999 | 1294 | 5.86 | 5.60 | 946 | 8.36 | 8.04 | 348 | 3.23 | 3.11 |
| 2000 | 1374 | 6.17 | 5.83 | 1027 | 9.02 | 8.59 | 347 | 3.19 | 3.02 |
| 2001 | 1383 | 6.17 | 5.70 | 1004 | 8.77 | 8.24 | 379 | 3.46 | 3.12 |
| 2002 | 1342 | 5.96 | 5.39 | 1025 | 8.92 | 8.19 | 317 | 2.87 | 2.56 |
| 2003 | 1508 | 6.67 | 5.91 | 1157 | 10.05 | 9.01 | 351 | 3.17 | 2.79 |
| 2004 | 1493 | 6.58 | 5.73 | 1123 | 9.73 | 8.61 | 370 | 3.32 | 2.84 |
| 2005 | 1530 | 6.72 | 5.73 | 1123 | 9.71 | 8.41 | 407 | 3.63 | 3.07 |
| 2006 | 1482 | 6.48 | 5.46 | 1116 | 9.63 | 8.24 | 366 | 3.24 | 2.71 |
| 2007 | 1579 | 6.88 | 5.64 | 1167 | 10.05 | 8.40 | 412 | 3.63 | 2.92 |
| 2008 | 1558 | 6.76 | 5.50 | 1162 | 9.99 | 8.27 | 396 | 3.47 | 2.77 |
| 2009 | 1504 | 6.51 | 5.23 | 1162 | 9.99 | 8.17 | 342 | 2.98 | 2.35 |
| 2010 | 1571 | 6.78 | 5.32 | 1194 | 10.26 | 8.20 | 377 | 3.27 | 2.52 |
| 2011 | 1579 | 6.80 | 5.27 | 1187 | 10.19 | 7.98 | 392 | 3.39 | 2.65 |
| 2012 | 1578 | 6.77 | 5.15 | 1216 | 10.42 | 8.05 | 362 | 3.11 | 2.36 |
| 2013 | 1511 | 6.46 | 4.85 | 1176 | 10.06 | 7.64 | 335 | 2.87 | 2.14 |
| 2014 | 1626 | 6.94 | 5.19 | 1231 | 10.52 | 8.00 | 395 | 3.36 | 2.48 |
| 2015 | 1492 | 6.35 | 4.69 | 1126 | 9.61 | 7.19 | 366 | 3.11 | 2.29 |
| 2016 | 1518 | 6.45 | 4.68 | 1146 | 9.78 | 7.21 | 372 | 3.15 | 2.24 |
| 2017 | 1525 | 6.47 | 4.64 | 1126 | 9.61 | 7.03 | 399 | 3.37 | 2.35 |
| 2018 | 1490 | 6.32 | 4.53 | 1122 | 9.58 | 6.95 | 368 | 3.10 | 2.21 |
| 2019 | 1560 | 6.61 | 4.68 | 1216 | 10.39 | 7.46 | 344 | 2.89 | 2.01 |
| 2020 | 1427 | 6.06 | 4.22 | 1077 | 9.23 | 6.51 | 350 | 2.94 | 2.03 |
| 2021 | 1465 | 6.27 | 4.29 | 1127 | 9.73 | 6.74 | 338 | 2.87 | 1.94 |
