# Supplementary material for: Trends in the incidence of the Epstein-Barr virus-associated malignancies extranodal NK/T-cell lymphoma and nasopharyngeal carcinoma in Taiwan
Source: PLoS One. 2024 Dec 31;19(12):e0315380. doi: 10.1371/journal.pone.0315380 (PMC11687711; doi:10.1371/journal.pone.0315380)
Supplement: S2 Table — (DOCX) [file pone.0315380.s002.docx]

S2 Table. Number of incidence cases, crude incidence rates (CRs) and age-standardized rates (ASRs) of patients with ENKTL between 2008 and 2021.

|  |  | Overall |  |  | Males |  |  | Females |  |
| --- | --- | --- | --- | --- | --- | --- | --- | --- | --- |
| Year | Number of  incidences | CR | ASR | Number of  incidences | CR | ASR | Number of  incidences | CR | ASR |
| 2008 | 65 | 0.28 | 0.23 | 46 | 0.40 | 0.34 | 19 | 0.17 | 0.13 |
| 2009 | 64 | 0.28 | 0.22 | 41 | 0.35 | 0.28 | 23 | 0.20 | 0.16 |
| 2010 | 49 | 0.21 | 0.16 | 31 | 0.27 | 0.21 | 18 | 0.16 | 0.12 |
| 2011 | 60 | 0.26 | 0.20 | 39 | 0.33 | 0.26 | 21 | 0.18 | 0.14 |
| 2012 | 78 | 0.33 | 0.24 | 48 | 0.41 | 0.31 | 30 | 0.26 | 0.18 |
| 2013 | 73 | 0.31 | 0.23 | 47 | 0.40 | 0.31 | 26 | 0.22 | 0.17 |
| 2014 | 56 | 0.24 | 0.16 | 36 | 0.31 | 0.21 | 20 | 0.17 | 0.11 |
| 2015 | 64 | 0.27 | 0.19 | 46 | 0.39 | 0.28 | 18 | 0.15 | 0.11 |
| 2016 | 64 | 0.27 | 0.18 | 41 | 0.35 | 0.24 | 23 | 0.19 | 0.13 |
| 2017 | 56 | 0.24 | 0.16 | 36 | 0.31 | 0.21 | 20 | 0.17 | 0.12 |
| 2018 | 62 | 0.26 | 0.17 | 40 | 0.34 | 0.23 | 22 | 0.19 | 0.13 |
| 2019 | 58 | 0.25 | 0.16 | 40 | 0.34 | 0.22 | 18 | 0.15 | 0.10 |
| 2020 | 59 | 0.25 | 0.16 | 37 | 0.32 | 0.20 | 22 | 0.19 | 0.14 |
| 2021 | 64 | 0.27 | 0.17 | 38 | 0.33 | 0.21 | 26 | 0.22 | 0.14 |
