# Supplementary material for: Trends in the incidence of the Epstein-Barr virus-associated malignancies extranodal NK/T-cell lymphoma and nasopharyngeal carcinoma in Taiwan
Source: PLoS One. 2024 Dec 31;19(12):e0315380. doi: 10.1371/journal.pone.0315380 (PMC11687711; doi:10.1371/journal.pone.0315380)
Supplement: S3 Table — (DOCX) [file pone.0315380.s003.docx]

S3 Table. Seroprevalence of EBV in Taiwan.

| Age (years) | 1984^1^ | 2007^2^ |
| --- | --- | --- |
| <1 | (<6 months) 38.4%  (6-12 months) 6.4% | 23.1% |
| 1 | 65.8% | 30.0% |
| 4 | 86% | 69.1% |
| 10-19 | 99% | 93% |

^1^ Tsai et al. 1989

^2^ Chen et al. 2007
